# Novel Concepts for HIV Vaccine Vector Design

**DOI:** 10.1128/mSphere.00415-17

**Published:** 2017-12-06

**Authors:** Quazim A. Alayo, Nicholas M. Provine, Pablo Penaloza-MacMaster

**Affiliations:** a Brigham and Women’s Hospital, Harvard Medical School, Boston, Massachusetts, USA; b Translational Gastroenterology Unit, Nuffield Department of Medicine, University of Oxford, Oxford, United Kingdom; c Department of Microbiology-Immunology, Feinberg School of Medicine, Northwestern University, Chicago, Illinois, USA; UMKC School of Medicine

**Keywords:** T cells, antibodies, human immunodeficiency virus, vaccines

## Abstract

The unprecedented challenges of developing effective vaccines against intracellular pathogens such as HIV, malaria, and tuberculosis have resulted in more rational approaches to vaccine development. Apart from the recent advances in the design and selection of improved epitopes and adjuvants, there are also ongoing efforts to optimize delivery platforms. Viral vectors are the best-characterized delivery tools because of their intrinsic adjuvant capability, unique cellular tropism, and ability to trigger robust adaptive immune responses. However, a known limitation of viral vectors is preexisting immunity, and ongoing efforts are aimed at developing novel vector platforms with lower seroprevalence. It is also becoming increasingly clear that different vectors, even those derived from phylogenetically similar viruses, can elicit substantially distinct immune responses, in terms of quantity, quality, and location, which can ultimately affect immune protection. This review provides a summary of the status of viral vector development for HIV vaccines, with a particular focus on novel viral vectors and the types of adaptive immune responses that they induce.

## Abstract (precis)

The unprecedented challenges of developing effective vaccines against intracellular pathogens such as HIV, malaria, and tuberculosis have resulted in more rational approaches to vaccine development. Apart from the recent advances in the design and selection of improved epitopes and adjuvants, there are also ongoing efforts to optimize delivery platforms.

## INTRODUCTION

Since Edward Jenner first demonstrated immunity to smallpox by inoculating a 13-year-old boy with vaccinia virus over 200 years ago, various vaccines effective against numerous microorganisms have been developed. However, the challenges of developing vaccines protective against intracellular pathogens such as human immunodeficiency virus type 1 (HIV-1) has necessitated the adoption of more rational approaches to vaccine design based on the systematic design of epitopes, the use of immunogenic adjuvants, and the selection of appropriate delivery platforms. Viral vectors belong to one such platform and have inherent adjuvant capability in the form of pathogen-associated molecular patterns that can trigger innate immune responses through their engagement of specific pattern recognition receptors. More importantly, different viral vectors may exhibit distinct cellular tropism, with specific innate and adaptive immune phenotypes that render them optimally poised for inducing immunological memory against particular pathogens. Although preexistent immunity is an important factor in vector selection, a better understanding of the immune correlates of protection will ultimately guide vaccine design. In this review, we will summarize the current status of viral vector-based HIV vaccines, highlighting the effects of various vectors on vaccine immunogenicity, safety, and efficacy. This will include a description of various virus vectors that have been used in HIV vaccine development, which include nonreplicating and replicating adenovirus type 5 (Ad5), alternative-serotype adenovirus, poxvirus, lymphocytic choriomeningitis virus (LCMV), cytomegalovirus (CMV), vesicular stomatitis virus (VSV), and attenuated immunodeficiency viruses.

## TARGETING SPECIFIC ARMS OF IMMUNITY AND ANTIGEN DESIGN: AN OVERVIEW

Given the complexities of HIV infection, it is increasingly clear that a successful vaccine must elicit multiple arms of adaptive immunity. One way to accomplish this goal is through the selection of distinct vaccine prime-boost platforms.

### Targeting both cellular and humoral arms.

Regimens involving heterologous viral vector priming followed by recombinant protein boosting represent one of the most promising strategies to induce potent cytotoxic T lymphocytes (CTL) and antibody responses. The RV144 trial, which is the most successful HIV vaccine trial to date, resulted in 31.2% protection from HIV infection and utilized a heterologous canarypox virus vector (ALVAC strain) priming-protein boosting approach (1). Correlates of risk analysis identified elevated V_1_V_2_-specific IgG antibodies as inversely associated with infection rates (2). Similarly, a subsequent study that utilized an adenovirus priming and protein boosting regimen achieved sterilizing protection in 40 to 50% of vaccinated macaques following repeated intrarectal challenges, and this protection was correlated with Env-specific antibody titers, as well as antibody-mediated effector functions (3). Other vaccine regimens utilizing DNA, protein, or viral immunizations that induce antibody and cellular immune responses have not translated into effective HIV vaccines. These include the VAX003 and VAX004 studies, which utilized gp120 monomer, and the HVTN 505 study, which utilized a DNA priming-Ad5 boosting regimen, as well as other Ad5-based studies, such as the STEP and Phambili trials (4).

### Targeting the cellular arm.

In the search for a potent HIV vaccine, vaccine regimens targeting mostly the CD8 T cell and/or the CD4 T cell components of the immune system have also been extensively explored. Viral vector-based vaccine regimens are capable of inducing robust antiviral CTL responses. The critical role of CD8 T cells in the control of HIV infection is highlighted by the strong association of robust CTL responses with control of infection in elite controllers and that experimental depletion of CD8 T cells in simian immunodeficiency virus (SIV)-infected macaques results in increased viral replication (5–7). Many of the viral vector-based vaccine modalities can induce virus-specific CTL responses that can reduce peak and/or set point viral loads following experimental SIV infection of rhesus macaques (8–11). However, such findings have not translated to the clinic, since all of these candidate HIV vaccines have failed to alter viral loads in vaccinated (and subsequently infected) individuals (1, 12, 13).

Furthermore, the important role of CD4 T cells in facilitating both the innate and adaptive immune systems has led to suggestions that vaccine modalities that preferentially induce CD4 T cell responses may be necessary for optimal vaccine-mediated protection. Indeed, it has been shown that CD4 T cells may be necessary for the effective control of certain diseases such as tuberculosis (14, 15), West Nile fever (16), and measles (17). Moreover, CD4 T cell help is required for the generation of memory CD8 T cells and high-affinity antibody responses (18–23), and poxvirus vectors, both the modified vaccinia virus Ankara (MVA) strain and the attenuated vaccinia virus strain derived from a Copenhagen vaccine strain that underwent multiple mutations in various open reading frames (NYVAC), appear to be particularly well suited for the induction of CD4 T cell responses (24–26).

However, prior studies have demonstrated that CD4 T cell-biased vaccines may be detrimental in the context of chronic viral infections (27, 28). In one of these studies, an SIV vaccine encoding a CD4 T cell epitope derived from Env resulted in increased SIV susceptibility and progression to AIDS (28). These data suggest that biased induction of CD4 T cells may be detrimental in the setting of HIV vaccination, which is not surprising, given that activated virus-specific CD4 T cells are a primary target of the virus (29). Interestingly, we showed that this phenomenon of “adding fuel to the fire” is generalizable to a distinct chronic viral infection model that does not primarily target activated CD4 T cells (27). Using the chronic LCMV mouse model, we showed that immunization with a vaccine that induces a CD4 T cell-biased response leads to fatal inflammatory disease following a challenge with chronic LCMV clone 13. This inflammatory disease induced by the CD4 T cell vaccine was prevented by transferring virus-specific CD8 T cells or antibodies, demonstrating that a balance of all arms of the adaptive immune response is critical for determining vaccine-induced protection in the context of chronic viral infection.

Lastly, it is important to mention that HIV exhibits a tremendous level of genetic heterogeneity, and therefore, a vaccine against this virus must be able to neutralize highly diverse viral strains. This challenge could be partially overcome by the *in silico* development of mosaic antigens that maximize immune responses to global circulating strains. A demonstration of this novel approach was reported in prior studies that demonstrate that mosaic antigens induce a greater depth and breadth of immune responses relative to consensus antigens (30, 31).

## VIRAL VECTORS

### Ad5 vectors.

With their ability to induce multiple arms of the immune system, viral vectors have been the most studied platforms in our search for an effective HIV vaccine. One of the earliest vectors, and thus the most studied, is Ad5. Ad5, a serotype C adenovirus, is one of the most immunogenic of the human adenoviral vectors. Several groups have shown that it induces potent humoral and cellular immunity in preclinical and clinical studies against a wide range of pathogens (32–35), as well as multiple tumor types (36, 37). Therefore, Ad5 has been used extensively in the pursuit of an HIV vaccine. Following the promising finding that Ad5 conferred protective immunity to a pathogenic SIV strain in macaques (38, 39), two clinical trials (STEP and Phambili) were set up to evaluate the ability of an Ad5 vaccine expressing HIV-1 subtype B Gag-Pol-Nef to elicit a protective cellular immune response against HIV-1 infection (12, 40). However, these trials were stopped before completion after interim analysis showed futility. Further analysis of the STEP trial also revealed a trend toward higher HIV acquisition among uncircumcized male vaccinees with preexisting Ad5 immunity (12). Another phase IIb efficacy trial (HVTN 505) that utilized priming with DNA and boosting with Ad5 expressing HIV-1 Gag-Pol-Nef antigens, as well as a modified HIV-1 Env transgene, also failed to show clinical efficacy (13).

These unexpected results of clinical trials with Ad5 have been suggested to be partly due to vaccine-induced T cell activation (41), but detailed analyses of the immunological properties of Ad5 suggest that other factors may also play a role. Studies with mice and nonhuman primates have demonstrated that the T cell responses elicited by Ad5 exhibit a partially exhausted T cell profile (42–45). Several groups have also shown that CD8 T cells induced by Ad5 are more terminally differentiated and exhibit impaired anamnestic expansion (43, 46, 47). Ad5-induced CD8 T cells also exhibit impaired central memory differentiation, evidenced by lower expression of the homeostatic survival marker CD127 and the lymphoid homing receptor CD62L than other Ad vector serotypes (42, 45). Importantly, the hallmark of exhausted CD8 T cells during chronic viral infection and cancer is the expression of inhibitory receptors such as programed cell death receptor 1 (PD-1), CTL antigen 4 (CTLA-4), T-cell immunoglobulin, mucin-3 (Tim-3), lymphocyte activation gene 3 (LAG-3), and the T-cell tyrosine-based inhibitory motif (ITIM) domain (TIGIT) (48). Intriguingly, we and others have shown that some of these inhibitory receptors, particularly PD-1, Tim-3, and CTLA-4, are permanently upregulated on Ad5-induced T cells (42, 43, 49). Those studies also demonstrated that although Ad5 induces a greater magnitude of transgene-specific CD8 T cells than other adenoviral vectors, Ad5-induced CD8 T cells are partially exhausted and show a reduced ability to secrete gamma interferon, tumor necrosis factor alpha, and interleukin-2. Recently, detailed transcriptional profiling of Ad5-induced transgene-specific CD8 T cells also showed an enrichment of transcriptomic signatures of anergy and exhaustion, further corroborating the phenotypic profile described above (49). Altogether, these features suggest that Ad5 induces a partially exhausted T cell response similar to what has been observed in chronic infection and cancer. It is important to note that despite these dysfunctional immune responses, Ad5-induced CD8 T cells still confer some protection in different challenge models, particularly in the absence of preexisting anti-Ad5 immunity (44).

The mechanisms underlying Ad5-induced immune exhaustion have not been fully elucidated. There are suggestions that multiple factors such as liver tropism (50, 51), antigen persistence or dose (42–44, 49), and impaired CD4 T cell help (45, 52) may play critical roles in the induction of T cell dysfunction.

### Alternative-serotype Ad vectors.

The above results with conventional Ad5 vectors have motivated the discovery of alternative-seroptype Ad vectors. Several vaccine vectors derived from rare human adenovirus types, as well as other species such as chimpanzees and rhesus monkeys, are being developed and evaluated against multiple pathogens (53–60). Of the rarer human adenovirus types, Ad26 and Ad35 are promising. Both utilize CD46 as their primary cellular receptor, unlike Ad5, which uses the coxsackievirus and adenovirus receptor (61, 62). Seroepidemiological studies assessing both novel vectors have demonstrated that they have significantly lower seroprevalence than Ad5 in many population groups (53, 63–65).

In terms of immunogenicity, prior studies have shown that Ad26 and Ad35 tend to be slightly less immunogenic than Ad5 (42, 45). However, the quality of immune responses induced by alternative-serotype Ad vectors or Ad5 vectors is distinct. Compared to Ad5, alternative-serotype Ad vectors induce a more potent innate immune response (66) and, as mentioned earlier, a more polyfunctional (and less exhausted) T cell response. Alternative-serotype Ad vectors also induce memory CD8 T cells with a long-lived central memory T cell phenotype and are thus better poised for robust T cell expansion following antigen reexposure. When used for priming in a heterologous prime-boost regimen with Ad35 or MVA boosting, Ad26 provided partial protective efficacy against multiple intrarectal challenges with two stringent strains of SIV (SIVmac251 and SHIV-SF162P3) in rhesus monkeys (9, 67). This protection seemed to be partly mediated by both functional neutralizing and nonneutralizing antibodies. Furthermore, in multiple clinical trials, Ad26 was shown to be safe and elicit polyfunctional humoral and cellular immune responses (68–70). Ad35 has also been shown to be safe and elicit potent immune responses, albeit smaller in magnitude than those elicited by Ad5 or Ad26 (42, 44, 53, 71). Because of their high expression, many immunoinhibitory pathways have been suggested to play a role in the regulation of Ad5-induced T cell exhaustion, including PD-1, CD200, CTLA-4, CD226, and LAG-3, all of which are decreased in Ad26-induced T cells, but their precise roles following immunization remain understudied (49).

Many groups are also developing chimpanzee- and rhesus macaque-derived serotypes because of their reduced seroprevalence (60, 72–75). In addition, chimpanzee Ad3 (ChAd3), which belongs to the same subgroup as Ad5 (subgroup C), induces CD8 T cells that are more polyfunctional than those induced by Ad5 (76). Importantly, the lower prevalence of preexisting anti-ChAd3 antibody in most populations may render this vector a suitable substitute for Ad5 (55). Overall, the quest for alternative-serotype Ad vectors has the potential to elucidate novel serotypes with low preexisting immunity, high immunogenicity, and distinct tropism, which could provide vaccinologists with a well-assorted toolkit to develop vaccines against HIV and other diseases.

### rLCMV vectors.

One limitation of the use of Ad vectors as vaccine platforms is their ability to elicit potent vector-specific neutralizing antibodies, which limits the efficacy of simple homologous boosting because of neutralization of the boosting homologous vaccine (77, 78). Although heterologous prime-boost vaccine regimens are often used to overcome this challenge, a single vector not hampered by preexisting humoral immunity is preferable, as it simplifies the prime-boost vaccine regimen and reduces the cost of production. To achieve this, a vector based on the arenavirus LCMV (which has been a main workhorse in basic immunology research because of its potent immunogenicity) was recently developed (79, 80). LCMV is a bisegmented negative-strand RNA virus that primarily infects rodents and has been widely used as a model to study cellular immunity (81, 82). Nonreplicating recombinant LCMV (rLCMV) vectors in which the LCMV glycoprotein (GP) gene is replaced with a vaccine transgene were shown to be highly immunogenic in mice and nonhuman primates, with the ability to target and induce dendritic cell activation and elicit persistent transgene-specific T cell responses (79, 83). The genetic absence of the GP gene in rLCMV vectors renders the virus replication defective, overriding concerns about potential LCMV-induced pathogenicity, which are especially considered in pregnant women and transplant recipients (84, 85). Interestingly, consecutive readministration of this vector as a homologous boost can lead to substantial anamnestic expansion of transgene-specific CD8 T cells and antibody responses without generating LCMV GP-specific neutralizing antibody responses (79, 80). Therefore, rLCMV provides an option for simpler, immunogenic homologous prime-boost vaccine modalities.

The unique ability of rLCMV to resist antibody neutralization is due to the absence of the gene encoding the LCMV GP in the vector. Moreover, the human seroprevalence of LCMV is reported to be <5%, compared to >20% for Ad26 and nearly 100% for Ad5 (53, 86–88). Efficacy studies have shown that an rLCMV-based vaccine protects mice against viral and tumor challenges and shows substantial protective efficacy in a model of SIV challenge of rhesus macaques when used for boosting following priming with Ad5 (83). Thus, rLCMV vectors constitute another novel tool in our armamentarium for developing protective vaccines against infectious diseases, as well as therapeutic cancer vaccines.

### Poxvirus vectors.

Poxvirus vectors, in the form of the canarypox virus-based ALVAC vector used in the RV144 trial, have proven to be the most clinically successful HIV vaccine vectors to date. The RV144 trial, in which ALVAC-HIV immunity was boosted with a recombinant glycoprotein 120 subunit vaccine (AIDSVAX B/E) and is colloquially referred to as the “Thai trial,” resulted in 31% protection from HIV acquisition associated with Env V_1_V_2_-specific IgG antibodies (1, 2, 89). Prevention of HIV acquisition was more striking during the first months after vaccination, when the levels of HIV-specific antibodies were highest. This degree of protection seemed to decrease over time and was associated with reduced antibody levels. Despite this rapid waning of antibody levels, the recent RV305 trial has demonstrated that long-lived memory B cells were induced in RV144 and a robust anamnestic antibody response can be recalled with a protein boost up to 8 years later (90). To build on the success of the RV144 trial, a new HIV vaccine trial (HVTN 702) was recently started in South Africa. The HVTN 702 regimen is a modification of the RV144 trial targeting the HIV subtype C prevalent in South Africa by using the same ALVAC vector for priming but with a different adjuvant (MF59) for Env protein boosting with the aim of generating a more robust and sustained antibody response.

In total, three additional poxvirus vectors have garnered significant attention as candidate HIV vaccine vectors. These vectors can be segregated into two groups, (i) orthopoxvirus-derived NYVAC (from the Copenhagen vaccine strain) and MVA and (ii) avipoxvirus-derived ALVAC (canarypox virus) and fowlpox virus. All four vectors are replication incompetent in mammalian cells (91–93). The ALVAC vector was particularly attractive because of the lack of preexisting antivector immunity in humans. In addition to the use of ALVAC in the RV144 trial and in the recently started HVTN 702 trial, there is also an increasing focus on MVA and NYVAC. In particular, MVA has gained considerable traction as a boosting vector following Ad vector priming, where such a regimen has induced partial protection from a neutralization-resistant SIV or SHIV challenge and significant reductions in viral loads (94, 95). Ad prime-MVA boost regimens have also shown promise against malaria (73), hepatitis C virus (96), and Ebola virus (97). In all cases, robust vaccine-elicited cellular immune responses were associated with protection from infection. A head-to-head comparison of NYVAC and ALVAC expressing HIV-1 clade C antigens using an immunization regimen based on RV144 showed modest superiority of the NYVAC-based regimen with regard to multiple measures of cellular and humoral immunogenicity (98). Whether these differences reflect true superiority of NYVAC-based vectors or are too modest to confer differences in protective efficacy remains to be rigorously tested. Further efforts are ongoing to modify and rationally select poxvirus vectors to better take advantage of the utility of poxvirus vectors as both priming and boosting vectors.

A growing literature has demonstrated that the different poxvirus vectors induce qualitatively different immune responses, akin to the observations described previously examining distinct Ad serotypes. Compiled comparisons of various poxvirus vectors demonstrate that ALVAC induces particularly strong antiviral and inflammatory responses in infected human cells and immunized macaques, compared to MVA, NYVAC, and fowlpox virus (11, 99–101). These comparisons identified MVA as the second most potent activator of innate immune stimulation, with NYVAC and fowlpox virus identified as the least stimulatory. In humans, MVA was more immunogenic than fowlpox virus, and a heterologous prime-boost regimen proved to be the most effective (24, 25). Phenotyping of T cell responses in macaques following MVA or NYVAC boosting of DNA-primed responses identified a mixed CD4 and CD8 T cell response induced by MVA, while NYVAC induced a predominantly CD4 T cell response (11). However, the overall magnitudes of the T cell responses were comparable. Finally, a comparison of NYVAC priming versus ALVAC priming of macaques showed increased CD4^+^ T cell and antibody responses in the NYVAC-primed animals (98). The paucity of direct comparisons of the different poxvirus vectors makes it difficult to interpret how the differences in innate immune activation by these vectors might translate into differences in induced adaptive responses. However, one study has demonstrated that MVA-derived antigens are more robustly presented by mammalian cells than canarypox virus-derived antigens. The immunogenicity and modest protective efficacy of poxvirus vectors mean they are one of the most promising vector modalities available. Overall, poxviruses are perhaps the most promising HIV vaccine vectors because of their ability to reduce HIV infection rates, and current efforts are aimed toward improving the durability of the antibody responses induced by these vectors.

### Replicating viral vectors.

Although any of the vectors discussed above could be developed as replicating vaccine platforms, a few new replicating vectors have garnered attention for their ability to induce immune protection against SIV in macaque studies. The impressive immune protection that is achieved by replicating virus vectors may be due to the generation of a special subset of CD8 T cells that are able to rapidly intercept the virus upon a challenge. Central memory CD8 T cells are long-lived, but they may exhibit a delay in reactivating their cytotoxic function following a viral challenge. On the other hand, effector memory CD8 T cells (which can be induced by replicating antigen) are short-lived but provide immediate cytotoxic function (102). Therefore, it has been proposed that the induction of effector memory CD8 T cells by certain replicating virus vectors may be critical for an HIV vaccine, since these responses are able to rapidly control initial infection foci before the virus becomes systemic (103). It is also important to mention that the duration of antigen stimulation can determine the levels of central memory versus effector memory CD8 T cell responses (104, 105), suggesting that viral vectors that replicate and persist for a long time may be better poised to induce effector memory CD8 T cells. Conversely, many replication-deficient adenoviral vectors, rLCMV vectors, and attenuated poxvirus vectors provide limited antigenic stimulation because of their rapid clearance by the host immune response and therefore elicit a biased central memory CD8 T cell response that preferentially localizes to lymphoid tissues (45, 47).

Of the various replication-competent vaccine vectors currently under development, recombinant human CMV (rhCMV) and replication-competent recombinant VSV (rVSV) (6, 106, 107) have been extensively characterized and are currently in clinical development.

### (i) rhCMV.

As a classical member of the herpesvirus family, rhCMV persists in the host, providing a constant source of antigen necessary for the maintenance of CD8 T cells with an effector memory phenotype. Similar to novel rLCMV vectors genetically lacking LCMV GP, the immunogenicity of rhCMV vectors is not limited by preexisting antivector immunity and they can therefore be used repeatedly, even in CMV-positive monkeys, to induce effector memory CD8 T cells (108). This property is particularly relevant because CMV has a seroprevalence of up to 90% in some populations (109). The absence of significant antibody responses following rhCMV immunization means that combination with other vaccine modalities that elicit protective humoral immune responses may be required to achieve optimal vaccine-mediated protection. However, impressive findings with the rhCMV platform in animal models (as high as 50% protection seen in animal studies) may be logistically challenging to translate to the clinic, given that human CMV infection can be associated with birth defects and severe complications in immunosuppressed or organ transplant patients (110, 111). However, there are ongoing efforts to generate a modified CMV vector that could circumvent these safety challenges. It is also important to mention that CD8 T cell responses elicited by rhCMV vectors have been shown to contradict conventional major histocompatibility complex (MHC) restriction paradigms. Effector CD8 T cells induced by such vectors can recognize MHC class II-restricted epitopes, which can result in more diverse immune recognition, especially at the sites of viral entry (112). These unconventional effector CD8 T cells may be important in both prophylactic and therapeutic HIV vaccines, as they provide a new pool of effector CD8 T cells that can target viruses that have escaped most conventional CD8 T cells.

### (ii) rVSV.

rVSV-based vectors have garnered renewed interest, given the recent highly successful phase III trials results of an rVSV-based vaccine for Ebola virus (113), another pathogen to which incredibly rapid immune responses are required for protection. Early testing of rVSV vectors showed promising prevention of disease progression in an intravenous SHIV (89.6P) infection model (114). However, concerns existed about the potential neurovirulence of the rVSV vector based on the original vector design (115). This is not surprising, given that VSV belongs to the rhabdovirus family, which also includes rabies virus. However, a redesigned, more attenuated, rVSV vector backbone that exhibits a lack of neurovirulence has been engineered (116, 117). Unexpectedly, the increased attenuation did not impair the immunogenicity of the transgene insert in mice and nonhuman primates, but protective efficacy has not been assessed. This revised construct has now been tested in phase I trials, where it displayed immunogenicity (6).

Efforts are ongoing to develop and test other replicating vectors that might also elicit rapid effector memory T cell responses. These efforts include recombinant replication-competent Ad4, Ad5, and Ad26 vectors (118–121), Sendai virus (122), herpes simplex virus (123), and NYVAC virus (124), many of which are in early-phase clinical and preclinical trials.

In addition, one of the most salient examples of immune protection in the SIV infection model is that induced by SIVΔnef (125). It has been shown that this attenuated SIV strain can confer substantial immune protection by a mechanism that is dependent on low-level antigen persistence that allows for the maturation of T and B cell responses (126–128). It is not completely clear what the contribution of SIVΔnef mutation is relative to immune phenotypic differentiation. Continuous SIVΔnef mutation after vaccination may provide epitope diversity to improve immune breadth and depth, but time-dependent maturation of T and B lymphocytes also seems to be critical. However, the use of attenuated immunodeficiency viruses poses reasonable concerns about the possibility of viral reversion, rendering this approach practically unfeasible in human trials.

As mentioned earlier, an advantage of replicating vectors is their enhanced antigen expression, which could potentially drive robust adaptive immune responses. High and permanent transgene expression can also be achieved by immunization with HIV-based lentiviral vectors, which integrate into the genome and provide persistent antigen expression, even in nondividing cells (129–131). One could conceptualize a model in which persistent transgene expression would be desirable to induce effector memory T cells that can quickly intercept a viral challenge at mucosal sites. Consistent with this, we have shown that priming with a replication-defective virus, followed by boosting with a highly replicating virus, is very effective at increasing the level of memory T cells. However, the reverse (priming with a highly replicating virus) results in immune exhaustion (132). Thus, the timing of prime-boost immunization is a critical aspect to consider in the next generation of prime-boost immunization regimens. Importantly, the safety consideration of using live replicating vectors is typically a factor that deters the use of these vectors in many clinical studies. Altogether, replicating viral vectors hold considerable promise as a distinct modality to elicit rapidly responding protective immunity, but the appropriate balance between immunogenicity and potential pathogenicity needs to be achieved.

## AAV vectors for antibody gene delivery.

Until now, we have discussed various vaccine vectors that are used for active immunization, which refers to the induction of the host immune response after exposure to an antigen. However, a main problem with HIV vaccine design is the difficulty of generating broadly neutralizing antibodies (bNAbs). This could be circumvented by cloning bNAb genes into viral vectors to directly induce the expression of bNAbs at the injection site, bypassing all of the steps that are required for the generation of bNAbs, including isotype switching, consecutive germinal center reactions, and somatic hypermutation. Recently, an adeno-associated virus (AAV) vector that constitutively expresses HIV-specific antibodies in a humanized mouse model of HIV infection has shown promise (133, 134). This approach of “engineering immunity” has proven successful in preventing HIV infection in humanized mice, but a caveat is that humanized mice do not express a functional immune system. Further studies with macaques showed that the AAV-vectored transgene is rapidly cleared by adaptive immune responses, substantially limiting the expression of the antibody genes (135, 136). Thus, a main goal of this approach is to modulate the pathways that mediate immune tolerance to prevent the rejection of the AAV vector, allowing for durable or permanent transgene expression.

## CONCLUDING REMARKS

The successful development of an HIV vaccine remains an unprecedented challenge. We have summarized here various virus vectors that are being developed in the quest to overcome this challenge (Table 1). One of the main challenges in HIV vaccine design is the high strain diversity of HIV and the difficulty of generating bNAbs by vaccination, which suggests the need for serial heterologous prime-boost immunizations. This may potentially preclude the possibility of a “single shot.” In addition, HIV can infect activated CD4 T cells that can be induced by vaccination or natural infection. Thus, a balance between protective antibody and cellular responses has been suggested to be one of the most critical aspects of HIV vaccine design (137, 138). Two main areas of difficultly remain for viral vectors. The first issue is preexisting vector immunity, and toward this end, rare-serotype vectors are constantly being characterized and developed. The second issue is how to best utilize vaccine vectors to elicit the appropriate adaptive immune responses (in terms of quantity, quality, and localization). As our knowledge of fundamental virology and immunology of vaccine vectors continues to expand, we hope to be able to translate this to successful HIV vaccine design through the rational engineering and refinement of promising vaccine modalities.

**TABLE 1:**
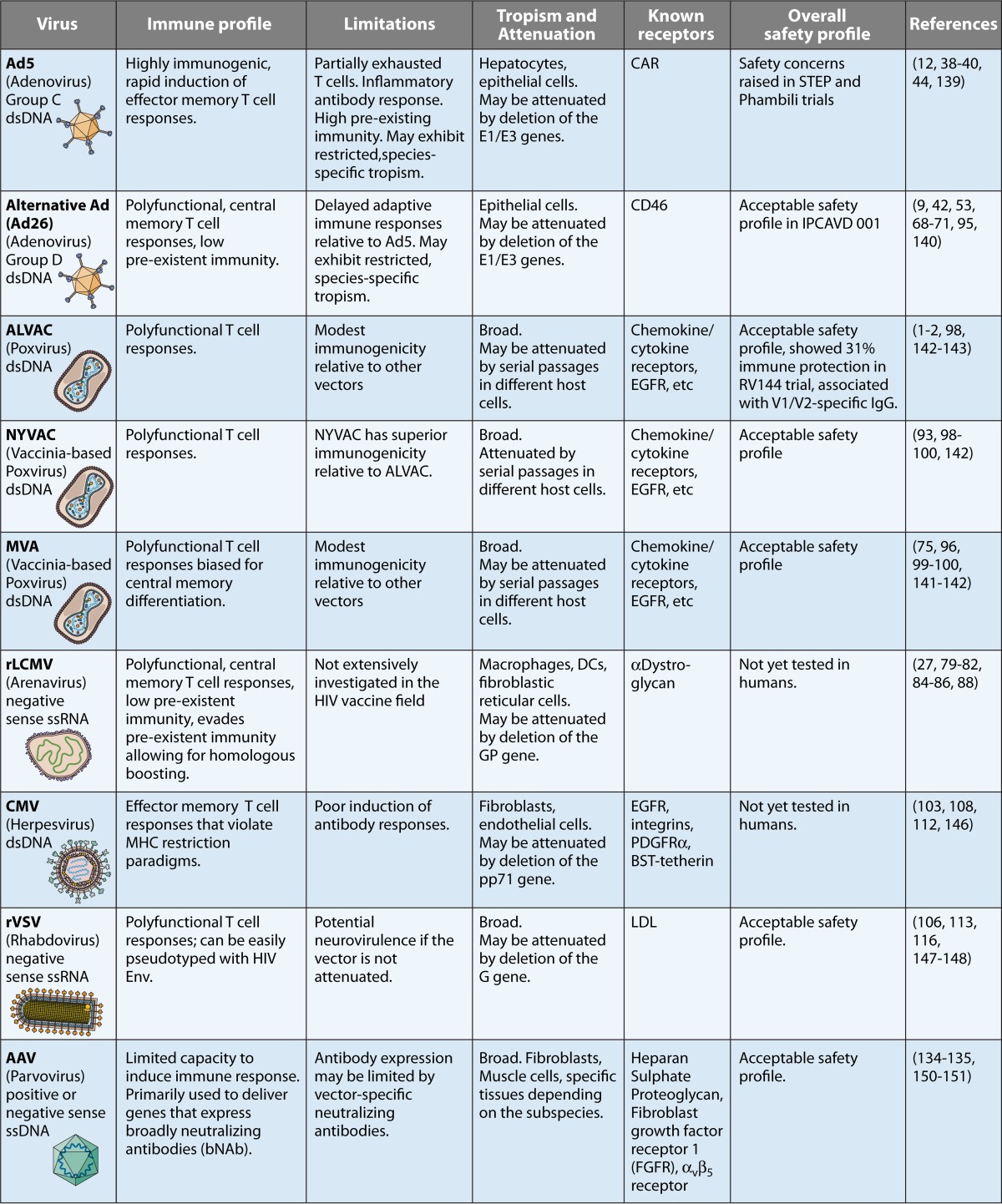
Summary of various vaccine vectors used in AIDS vaccine development
